# Monitoring access to nationally commissioned services in England

**DOI:** 10.1186/1750-1172-7-85

**Published:** 2012-10-30

**Authors:** Suzanne Coles, Kate Haire, Tom Kenny, Edmund G Jessop

**Affiliations:** 1 National Specialised Commissioning Team, 2nd Floor Southside 105 Victoria Street, London, SW1E 6QT, UK

**Keywords:** Specialised services, Monitoring, Access, Commissioning, Systematic component of variation, National case load

## Abstract

**Background:**

For over 20 years, the National Health Service in England has run a system of national planning for highly specialised healthcare services. The aim is to ensure that very rare diseases are treated, and very complex procedures performed, in only a few centres, each of which maintains a volume high enough to maintain excellent outcomes. The commissioning strategy for the provision of these national services in England is strongly centralising. Centralising does however create a duty to ensure that patients distant from the treatment centres are not thereby disadvantaged. The commissioning process ensures sufficient capacity to treat the entire national caseload of clinically eligible patients. The aim of this paper is to apply the Systematic Component of Variation (SCV) to study access to services commissioned by the National Specialised Commissioning Team (NSCT) in England. The discussion focuses on the potential explanations for a high level of systematic variation between areas and on the use of the SCV to support the monitoring and development of these nationally commissioned services.

**Method:**

Data from nationally commissioned services for the year ending 2011 were received from treating hospital. Mid year age and sex appropriate population estimates were then obtained to provide denominator data. Data were analysed at the geographic level of strategic health authority.

**Results:**

30 services met all requirements for analysis. There is no apparent relationship between SCV and number of locations from which the service is provided. On inspection high SCV is more common among recently commissioned services.

**Discussion:**

The importance of the SCV lies in its ability to support the development of highly specialised services. Once the random variation has been accounted for, the reasons for a systematic component can be explored. While no absolute cut- off exists, the SCV can be used to gauge and explore services that are potentially not covering the national caseload. The reason for a high SCV may not be immediately apparent; thus the SCV can aid those responsible for commissioning the service to seek potential explanations and identify improvements.

**Conclusion:**

We have reviewed spatial variation in access to a set of highly specialised services in England. On inspecting our results, we believe that they suggest that equity of access can usually be achieved at about five years after establishing a service, and this is not dependent, within the geography of England, on the number of centres designated.

## Introduction

For over 20 years, the National Health Service in England has run a system of national planning for highly specialised healthcare services. In its current form, the National Specialised Commissioning Team (NSCT) commissions a set of around 60 highly specialised services for the National Health Service in England [1]. Generally speaking, these are services that affect fewer than 500 people across England or involve services where fewer than 500 highly specialised procedures are undertaken each year. The aim is to ensure that very rare diseases are treated, and very complex procedures performed, in only a few centres, each of which maintains a volume high enough to maintain excellent outcomes. Hence the commissioning strategy is strongly centralising. Nationally commissioned services are funded from a central budget so access to the service is free to the patient and to the local health economy.

Centralising does however create a duty to ensure that patients distant from the treatment centres are not thereby disadvantaged. The commissioning process ensures sufficient capacity to treat the entire national caseload of clinically eligible patients.

Since there will inevitably be some random variation in access to services between areas, a statistical measure of variation is needed to identify if the variation is more (or less) than a random effect. The Systematic Component of Variation (SCV) performs well [2,3] and has previously been used to study spatial equity of access to local services in the National Health Service in England [4,5]. The SCV metric partitions observed variation between areas into a random component, using a Poisson assumption, and a systematic component.

The aim of this paper is to apply the SCV to study access to services commissioned by the NSCT in England. Access is a complex construct; in this paper we use service use as a measure for access. The discussion focuses on the potential explanations for a high level of systematic variation between areas and the use of the SCV to support the monitoring and development of these nationally commissioned services. There is a prior expectation that geographical variation in access will decrease as services are taken into national commissioning and that the longer a service has been commissioned nationally the more uniform the coverage, with the expectation that they would have a lower SCV. Without national commissioning local budget holders often refuse funding (and hence access) to these high cost services; national commissioning removes this barrier. This inequity, known in England as ‘postcode lottery’ is an important factor in ministers’ decisions to take a service into national commissioning. A national service also attracts publicity among doctors and patients, increasing the likelihood of referral to an expert centre.

## Method

Numerator data (counts of patients, caseload or procedures as appropriate) for the year ended 31 March 2011 were submitted by treating hospitals from local databases. ‘Patients’ are patients new to the service seen for the first time during the year in question. ‘Caseload’ is patients seen face-to-face by the service during the year ended 31 March 2011; each patient is only counted once. The data were coded by Primary Care Trust of residence. Data for transplants were kindly supplied by NHS Blood and Transplant as they hold these data.

Denominator data (mid year population estimates for 2010) were obtained from the Office for National Statistics [6]. For conditions affecting only children or only adults or only one sex appropriate age- and sex- specific denominator populations were used.

Data were analysed at the geographical level of Strategic Health Authority. The Strategic Health Authorities cover diverse populations and divide England into ten areas with total populations ranging from 2.6 million to 7.8 million.

With ten units of analysis it was thought that a service with a numerator count less than 30 would have data too sparse for sensible analysis. For this reason these services were excluded from the analysis. In addition services that were unable to produce a full dataset from each centre where the service was delivered were also excluded. Services where the available data did not meet Poisson assumptions (e.g. consisted of total attendances, including repeat attendance by the same individual) were also excluded. Services for young people in secure mental facilities represents an example of this.

The start date for the service is defined as the date on which national commissioning of the service commenced. The number of locations is defined as the number of places (cities) in which the service is provided, but London is counted as one location even if there are two or more providers in London.

The calculation for the SCV is easily executed using a spreadsheet, which is available from the corresponding author on request.

## Results

Of the 51 national services commissioned at 31 March 2010, 10 were excluded because the numerator for analysis was less than 30. A further 11 services were excluded either for lack of a full dataset from all centres, or because the available data did not meet Poisson assumptions. This left 30 services available for analysis.

Results are shown in the Table 1. There is no apparent relationship between SCV and number of locations from which the service is provided. Eleven services had an SCV of 20 or more, of which 8 have been operating as nationally commissioned services for 5 years or less.

**Table 1:** Geographic variation in nationally commissioned services ordered by year service commissioned

**Service**	**Start year**	**Metric**	**Number**	**SCV**	**Highest rate per million**	**Lowest rate per million**	**Number of locations**
Choriocarcinoma	1984	Patients	131	4	9.2	1.1	2
**Specialist paediatric liver disease**	**1985**	Caseload	**3405**	**25**	**671.9**	**100.4**	**3**
Severe combined immune deficiency	1993	Patients	36	0	6.6	0.0	2
Elective liver transplant (adult)	1995	Transplants	371	1	12.0	6.2	6
Elective liver transplant (child)	1995	Transplants	49	11	8.2	0.0	3
**Severe intestinal failure**	**1998**	Caseload	**318**	**70**	**17.8**	**0.5**	**2**
Amyloidosis	1999	Caseload	409	4	11.3	4.9	1
Pseudomyxoma peritonei surgery	2000	Patients	111	5	5.1	1.0	2
Pulmonary thrombendarterectomy	2000	Patients	106	9	4.5	0.6	1
Lung transplant (adult)	2002	Transplants	135	3	5.9	1.3	5
Heart transplant (adult)	2002	Transplants	71	8	3.5	0.5	5
Heart transplant (child)	2002	Transplants	31	8	6.6	0.0	2
Epidermolysis bullosa	2002	Caseload	1102	17	44.2	8.8	2
**Pancreas transplant**	**2004**	Transplants	**171**	**27**	**10.4**	**1.4**	**5**
Lysosomal storage disorder (adult)	2005	Caseload	917	0	27.2	19.2	4
Primary malignant bone tumour	2005	Patients	495	13	20.0	6.0	5
Lysosomal storage disorder (child)	2005	Caseload	553	16	102.4	26.8	4
Complex tracheal surgery	2006	Patients	51	15	9.7	1.0	1
**Congenital hyperinsulinism**	**2006**	Caseload	**458**	**34**	**95.4**	**8.7**	**2**
Pulmonary hypertension (child)	2007	Caseload	279	8	48.4	17.1	1
**Severe obsessive compulsive disorder**	**2007**	Patients	**116**	**50**	**6.7**	**0.2**	**1**
Gender identity development	2009	Patients	132	5	21.9	6.2	1
**Complex Ehlers Danlos syndrome**	**2009**	Patients	**107**	**26**	**4.9**	**0.4**	**2**
**Complex neurofibromatosis type I**	**2009**	Caseload	**700**	**74**	**32.2**	**1.3**	**2**
**Chronic pulmonary aspergillosis**	**2009**	Caseload	**225**	**268**	**31.0**	**0.0**	**1**
Xeroderma pigmentosum	2010	Caseload	49	8	2.0	0.2	1
Biedl Bardet syndrome (adult)	2010	Caseload	111	11	4.4	0.5	2
**Complex neurofibromatosis type II**	**2010**	Caseload	**513**	**20**	**16.0**	**2.2**	**4**
**Cryopyrin associated periodic syndrome**	**2010**	Caseload	**37**	**31**	**1.7**	**0.0**	**1**
**Neuromyelitis optica**	**2010**	Caseload	**193**	**132**	**13.5**	**0.2**	**2**

Table ordered by start date for national commissioning. Services with SCV 20 or greater are shown in bold.

‘Patients’ means patients seen for the first time by the service; ‘caseload’ means patients (new and old) seen face-to-face by the service during the reporting period. ‘Transplants’ means the number of transplants.

‘Number’ is the number of patients, caseload or transplants. ‘Rate per million’ is the highest (lowest) rate per million among the 10 geographical areas analysed.

## Discussion

The SCV gives an estimate of the relative systematic component of variation between two areas by subtracting the random component of variance from the estimated of the total variance [3]. Therefore the calculation of the SCV gives an estimation of amount variation for a given set of standardised rates. This allows a comparison between a set of such rates to be made; in this paper the set is defined by services that are commissioned by the NSCT. Unlike a p-value which has an accepted statistical value, the SCV has no absolute interpretation but in previous work a cut-off of 16 has been suggested in one paper as indicative of high variability [5]. In the present study, we took an arbitrary value of 20 to indicate a high level of systematic variation.

There are several potential explanations for a high level of systematic variation between areas.

The first is that disease incidence or prevalence is not uniform. This may apply to very rare diseases which are strongly genetic. Neurofibromatosis Type 1 may be an example of this. Approximately 50% of cases of Neurofibromatosis Type 1 have an identifiable inherited cause [7], but these cases often occur in geographical grouping.

Secondly, a newly developed service will in its early years tend to treat the local catchment first. Variation will decrease as the service develops full national coverage. Three of the 17 services commissioned before 2006 show high systematic variation compared to 8 of the 13 services commissioned in 2006 or later. This suggests that the act of national commissioning provides a mechanism to reduce variation in access to specialised services. Almost all services taken into national commissioning arrangements have been running before national commissioning and attempting to achieve national coverage, but the high variation at onset and in the early years suggests very patchy coverage prior to national commissioning.

Thirdly, patients remote from the treatment centre may be disadvantaged in their access to highly specialised services. Patients with very rare conditions almost always express a willingness to travel any distance to see treatment teams who are thoroughly knowledgeable about their condition, particularly if the visits are not too frequent (annual review or one-off procedures). Nevertheless distance effects may occur either because of the burden of travel, or because of lesser awareness among clinicians in remote centres of either the disease itself or of the benefits of treatment. The problem of awareness is likely to apply particularly to the newly commissioned services.

This mechanism will also apply if referral criteria for the highly specialised service are not completely clear. We believe that this is the mechanism for the inequity in the services for severe intestinal failure. In this service subjective judgements are required among referring clinicians about degrees of severity and hence about which patients to refer.

Finally variation may be a data artefact if the quality of data are poor. Returns from treatment centres for nationally commissioned datasets are, unlike routine NHS data sources, almost all made from bespoke databases and validated by the treating clinicians. Hence we believe that the quality of the numerator data used in this study is high.

### Application of the SCV

The importance of the SCV lies in its ability to support the development of highly specialised services. Once the random variation (which will exist to a certain extent in the majority of services) has been accounted for, the reasons for a systematic component can be explored. While no absolute cut- off exists, it can be used to gauge and explore services that are potentially not covering the national caseload. The reason for a high SCV may not be immediately apparent thus the SCV can aid those responsible for commissioning the service to seek potential explanations and indentify improvements. The calculation of the SCV should be encouraged by those responsible for commissioning highly specialised services and incorporated the into evaluation process. Policy responses to high SCV include education and awareness campaigns in low access areas, outreach clinics and occasionally development of a new expert centre.

It is not always obvious what statistic to count if the aim is to ensure equity of access. Indeed equity of outcome is what we really want to achieve. However that data are very difficult to capture. Within equity of access, we may choose to count all contacts with the service, or only those which result in a face-to-face consultation, or only those in which the patient turns out, after assessment, to meet the service definition.

### Limitations

There are potential limitations around the statistical measurement of the SCV as it has no conventionally accepted cut off point. Therefore all we can state is the size of the ratio rather than an absolute measure. The SCV is not currently routinely used and therefore may be less widely accepted as a routine measure of spatial equity. However with greater awareness of its potential application this situation may change.

## Conclusion

We have reviewed spatial variation in access to a set of highly specialised services in England. On inspecting our results we believe that they suggest that equity of access can usually be achieved at about five years after establishing a service, and this is not dependent, within the geography of England, on the number of centres designated.

### Ethical review

Ethical review not needed (analysis of anonymised datasets).

## Competing interests

The authors declare that they have no competing interests.

## Authors’ contributions

Original idea formulated by EGJ all authors contributed to and reviewed final manuscript.
